# Autologous Matrix-Induced Chondrogenesis (AMIC) and Microfractures for Focal Chondral Defects of the Knee: A Medium-Term Comparative Study

**DOI:** 10.3390/life11030183

**Published:** 2021-02-25

**Authors:** Filippo Migliorini, Jörg Eschweiler, Nicola Maffulli, Hanno Schenker, Alice Baroncini, Markus Tingart, Björn Rath

**Affiliations:** 1 Department of Orthopedics and Trauma Surgery, University Clinic Aachen, RWTH Aachen University Clinic, 52064 Aachen, Germany; migliorini.md@gmail.com (F.M.); joeschweiler@ukaachen.de (J.E.); hschenker@ukaachen.de (H.S.); alice.baroncini@gmail.com (A.B.); mtingart@ukaachen.de (M.T.); Bjoern.Rath@klinikum-wegr.at (B.R.); 2 Department of Medicine, Surgery and Dentistry, University of Salerno, Via S. Allende, 84081 Baronissi, Italy; 3 School of Pharmacy and Bioengineering, Keele University School of Medicine, Thornburrow Drive, Stoke-on-Trent ST5 5BG, UK; 4 Centre for Sports and Exercise Medicine, Barts and the London School of Medicine and Dentistry, Queen Mary University of London, Mile End Hospital, 275 Bancroft Road, London E1 4DG, UK; 5 Department of Orthopedics, Klinikum Wels-Grieskirchen, A-4600 Wels, Austria

**Keywords:** autologous matrix-induced chondrogenesis (AMIC), chondral defects, knee

## Abstract

Introduction: The potential of autologous matrix-induced chondrogenesis (AMIC) to restore unipolar focal chondral defects of the knee is promising. However, the outcome compared to microfracturing (MFx) for certain defect sizes (2–3 cm^2^) is still uncertain. Therefore, the present study compared primary isolated AMIC versus MFx in a cohort of patients with borderline sized focal unipolar chondral defects of the knee at midterm follow-up. **Methods:** Patients with chondral defects of the knee who underwent AMIC or MFx were compared. An arthroscopic approach was used for MFx, and a minimally invasive parapatellar arthrotomy for AMIC. For those patients who underwent AMIC, a collagen membrane was used with fibrin glue. The patients answered independently: Visual Analogic Scale (VAS), Tegner Activity Scale, International Knee Documentation Committee (IKDC), and the Lysholm scores. **Results:** A total of 83 patients with a mean age of 30.2 and body mass index (BMI) of 26.9 kg/m^2^ were recruited. Of them, 33.7% (28 of 83) were women, and 55.4% (46 of 83 patients) had defects in the right knee. The mean length of symptoms before surgery was 43.3 months. The mean size of the defect was 2.7 cm^2^. The mean length of follow-up was 42.1 months. No difference was found in terms of symptoms and follow-up length, mean age and BMI, mean size of defect, sex, and side. The AMIC cohort reported greater IKCD (*p* > 0.0001), Lysholm (*p* = 0.002), VAS (*p* = 0.01), Tegner (*p* = 0.004) scores. The AMIC cohort reported lower rate of failure (*p* = 0.005) and revision surgery (*p* = 0.02). No difference was found in the rate of arthroplasty (*p* = 0.2). No delamination or hypertrophy were detected. **Conclusion:** AMIC demonstrated superiority over MFx for focal unipolar chondral defects of the knee. At approximately 40 months follow-up, the IKDC, Lysholm, and VAS scores were greater in the AMIC group. Patients treated with AMIC also demonstrated a higher level of sport activity, and lower rates of failure and revision surgeries.

## 1. Introduction

Focal chondral defects of the knee are common and are detected in up to 72% of patients undergoing knee arthroscopy [1,2]. Symptomatic chondral defects impair the quality of life and sporting activity level of the affected patients [3,4]. The alymphatic and hypocellular hyaline cartilage along with its low metabolic activity account for its poor regenerative capabilities [5,6,7]. Acute chondral injuries usually do not result in a restitutio ad integrum, rather in the production of fibrocartilage, and residual chondral defects are common [8,9]. Treating these patients is challenging and controversial [10,11]. Generally, isolated microfractures (MFx) have been proposed for chondral defects up to 2.5 cm^2^ [11,12,13,14,15]. For larger defects, several surgical strategies are available. Osteochondral allograft and/or autograft transplantation (OAT) and autologous chondrocyte implantation (ACI) have been widely performed in bigger defects [16,17,18]. However, the need of a harvesting site, two stage surgeries, or cell culture and expansion, have encouraged researchers to develop less labour-intensive strategies [19,20,21]. To overcome these problems, in 2005, Behrens et al. [22] firstly described an enhanced microfractures technique, which developed into the autologous matrix-induced chondrogenesis (AMIC) procedure. AMIC does not necessitate harvesting any autologous tissue to extract and expand chondrocytes [4,23]. Moreover, AMIC is performed in a single-session surgery, exploiting the potential of bone marrow-derived mesenchymal stem cells (BM-MSCs) [24,25]. Thus, AMIC quickly gained ground in the field of cartilage defect regeneration [26]. 

To the best of our knowledge, only three studies have compared AMIC versus MFx in knee with chondral defects [27,28,29]. However, no previous study has analysed the outcome of tibio- and patellofemoral lesions in a separate fashion, nor have primary procedures and revision settings been considered separately. Furthermore, the indications for borderline sized defects (2.2 to 2.8 cm^2^) are debated. The present study compared primary isolated AMIC versus MFx in a cohort of patients with focal chondral defects of the femorotibial joint of the knee at midterm follow-up.

## 2. Material and Methods

### 2.1. Study Design

**Figure 1:**
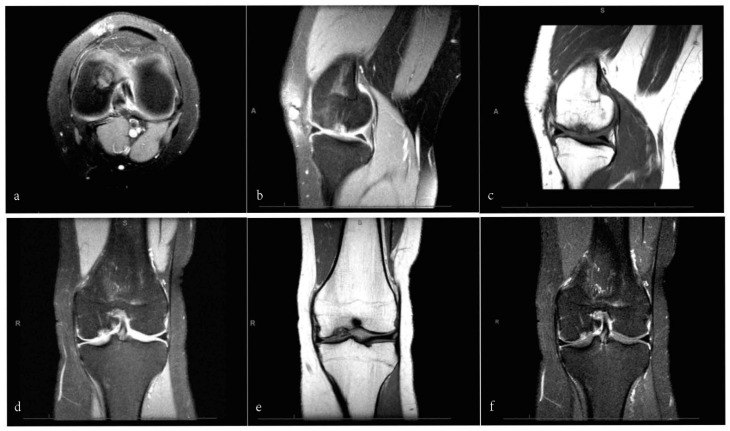
MRI sequences of a focal chondral defect of the medial femoral condyle in a 28-year-old male patient: axial (**a**) and sagittal (**b**) views using a proton density turbo spin-echo SPIR sequence; sagittal (**c**) and coronal (**e**) views of T1-weighted SPIR sequence; coronal view of proton density turbo spin-echo (**f**) and with SPIR sequence (**d**).

The present study was performed according to the Strengthening the Reporting of Observational Studies in Epidemiology (STROBE) [30]. This study was conducted in the Department of Orthopaedic Surgery of the University Hospital RWTH Aachen, Germany, between 2012 and 2020. Patients undergoing primary isolated AMIC or MFx for borderline sized unipolar focal chondral defects of the knee were examined, and their suitability to participate in this study was evaluated. The inclusion criteria were (1) symptomatic chondral defect, (2) single focal defect sized 2 to 3 cm^2^, (3) magnetic resonance imaging (MRI) evidence (Figure 1), (4) being able to understand the nature of the treatment and the study. The exclusion criteria were (1) kissing lesions, (2) bilateral lesions, (3) multiple lesions, (4) previous knee surgery, (5) any bone disease, (6) varus or valgus deformity, (7) grade II to IV according to the Kellgren and Lawrence grading system [31]. In case of suspected varus/valgus axial deformities, we obtained plain weightbearing radiographies to evaluate the Q-angle. Suitable patients received information about pros and cons of both techniques, and if they consented to participate in this study, they were free to decide their own treatment. Finally, in 2020, patients were invited to participate in our investigation. The present study was approved and registered by the ethics committee of the RWTH University of Aachen (project ID EK 438-20) and conducted according to the principles expressed in the Declaration of Helsinki. All patients were able to understand the nature of their treatment and provided written consent to use their clinical and imaging data for research purposes.

### 2.2. Surgical Technique

All the surgeries were performed in the same fashion by two experienced surgeons (B.R. and M.T.) according to a previous report [32]. Briefly, preliminary diagnostic arthroscopy was performed through standard anteromedial and anterolateral portals. After debridement of the chondral defect to achieve stable cartilage borders, we performed microfractures of 4 mm depth in a full-arthroscopic fashion. For those patients who underwent AMIC, a minimally invasive parapatellar arthrotomy was performed. Debridement and curettage of the non-viable tissues surrounding the lesion was then performed. In cases of subchondral bone defect (e.g., osteochondral defect, osteochondritis dissecans), non-vital bone was removed, and the defect was filled with autologous cancellous bone graft harvested from the ipsilateral iliac crest. A type I/III resorbable collagen membrane (from 2012 to 2016: Chondro-Gide, Geistlich Pharma AG, Wolhusen, Switzerland; from 2016 to 2020: Cartimaix, Matricel, Herzogenrath, Germany) was trimmed to slightly undersize the defect to avoid displacement. The membrane was hydrated in a saline solution and placed into the lesion. Fibrin glue was used to secure the membrane into the defect. The stability of the membrane was checked by repeatedly flexing and extending the knee under direct vision. Irrespective to the treatment allocation, patients received the same post-operative rehabilitation. Continuous passive motion up to 90° on the operated knee started 12 h after surgery to minimise the risk of intra-articular adhesions, along with isometric contractions of the quadriceps and active flexion of the ankle to encourage lower extremity circulation. For the first 6 post-operative weeks, patient ambulation was allowed, with maximal 15 kg of weightbearing using 2 elbow crutches. Over the following 2 weeks, only half body weightbearing was allowed. Starting from 10 weeks, full weightbearing was allowed. 

### 2.3. Outcomes of Interest

On admission, age, gender, side, area of defect, additional autologous cancellous bone grafting, body mass index (BMI), symptom duration prior to surgery, and length of hospital stay were recorded. Following written informed consent, the patients performed the following scores: Visual Analogic Scale (VAS), Tegner Activity Scale, International Knee Documentation Committee (IKDC), and the Lysholm scores. The Magnetic Resonance Observation of Cartilage Repair Tissue (MOCART) score was assigned by a blinded radiologist not involved in the clinical management. Data concerning the rate of complications (failure, revision, arthroplasty, delamination, hypertrophy) and additional procedures were also collected. Failure was defined as persistent pain that negatively affected the quality of life and limited participation to recreational activities. A subgroup analysis was performed to investigate differences between patients receiving cancellous bone grafting and those undergoing an isolated chondral procedure. A further subgroup analysis was conducted to investigate whether the 2 membranes (Chondro-Gide, Cartimaix) provided different outcomes.

### 2.4. Statistical Analysis

All statistical analyses were performed using the software IBM SPSS version 25. Continuous data were analysed using the mean difference (MD), while for dichotomic data, the odds ratio (OR) effect measures were calculated. The confidence interval was set at 95% in all the comparisons. The *t*-test and χ^2^ tests were performed, with values of *p* < 0.05 considered statistically significant.

## 3. Results

### 3.1. Recruitment Process

**Figure 2:**
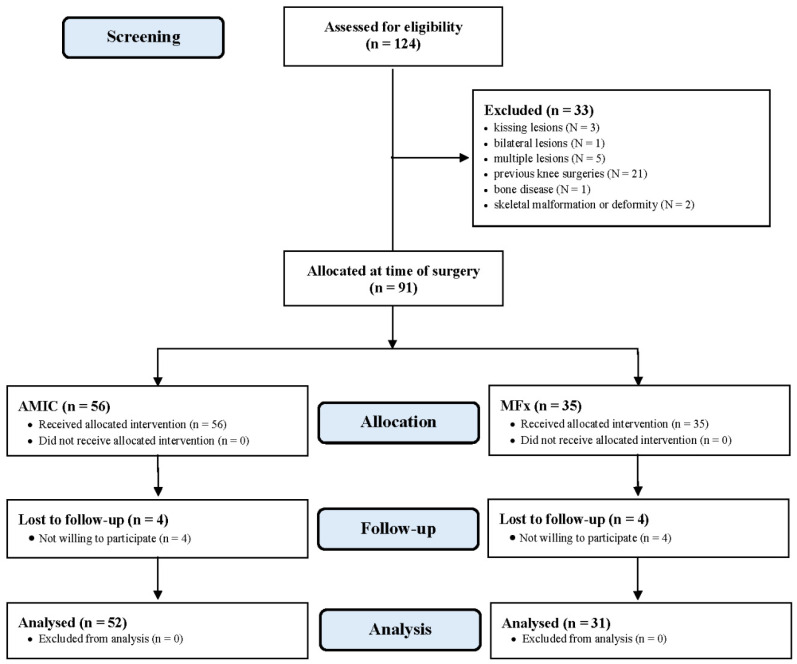
Diagram of the recruitment process.

A total of 124 patients were initially screened. Of them, 33 were not eligible due to kissing lesions (*N* = 3), bilateral lesions (*N* = 1), multiple lesions (*N* = 5), previous knee surgeries (*N* = 21), bone disease (*N* = 1), or skeletal malformation or deformity (*N* = 2). A total of 91 patients were available and operated: 56 AMIC and 35 microfractures. At the last follow-up, four patients in the AMIC group and four in the microfractures group were not available. The eight patients who did not attend the last follow-up were contacted telephonically and declared themselves satisfied but unavailable to attend assessment for geographical reasons. Eventually, 83 patients were enrolled in the present study: 52 underwent AMIC, and 31 MFx (Figure 2). 

### 3.2. Patient Demographics

We analysed data from the 83 patients who completed the study. Of them, 33.7% (28 of 83) were women, and in 55.4% (46 of 83 defects) the lesion was located on the right side. The mean age was 30.2, and the mean BMI was 26.9 kg/m^2^. The mean duration of symptoms before surgery was 43.3 months. The mean size of the defect was 2.7 cm^2^ (1.9 to 3.1). The mean length of the follow-up was 42.1 months. The MFx group had a shorter duration of the hospitalisation compared to the AMIC cohort (*p* = 0.03). No difference was found in terms of symptoms and follow-up length, mean age and BMI, mean size of defect, sex, and side (Table 1).

### 3.3. Outcomes of Interest

The AMIC cohort reported greater IKCD (*p* = 0.007), Lysholm (*p* = 0.02), VAS (*p* = 0.008), and Tegner (*p* < 0.0001), while the MOCART score was similar (*p* = 0.7) (Table 2).

### 3.4. Complications

The AMIC cohort reported lower rate of failure (*p* = 0.005) and revision surgery (*p* = 0.02). No difference was found in the rate of conversion to arthroplasty during the duration of the follow-up (*p* = 0.2). No delamination or hypertrophy were detected (Table 3).

### 3.5. Subgroup Analysis

No statistically significant difference was found in term of PROMs and complications between the AMIC subgroup that received cancellous bone grafting compared to those that received only the chondral procedure, and between those that received the Chondro-Gide and the Cartimaix membranes. 

## 4. Discussion

The main finding of the present study was that AMIC demonstrated superiority over MFx for focal unipolar chondral defects of the knee of an average area of 2.7 cm^2^. At approximately 40 months follow-up, IKDC, Lysholm, and VAS were greater in the AMIC group. Patients treated with AMIC also demonstrated a higher level of sporting activity, along with lower rates of failure and revision surgery. The MOCART score detected no morphological difference in the cartilage at final follow-up. Similarity was found between patients who received cancellous bone grafting compared to those who received only the chondral procedure, and between those who received the Chondro-Gide and the Cartimaix membranes. 

For chondral defect of the knee up to 2.5 cm^2^, MFx is indicated [11,12,13,14,15], and AMIC has been proposed for larger defects [4,23,24,25]. The patients included in the present study presented a mean defect area of 2.7cm^2^, slightly larger than what classically considered as the upper limit for MFx. The treatment of patients with borderline defect size is debated. Given its rapidity, the avoidance of arthrotomy, and the quick recovery time, MFx is often preferred in patients with borderline defect sizes. However, our results demonstrated that for borderline lesions, AMIC provided better outcomes, justifying the procedure. We were able to identify three studies that compared AMIC versus MFx for knee chondral defects [27,28,29]. Chung et al. [29] compared AMIC versus MFx on 64 patients. Although the overall values of IKDC and VAS were significantly better in the AMIC group at two years follow-up, the differences were not statistically significant. However, they performed the surgeries in a cohort of patients with a mean defect size of 1.3 cm^2^ in the AMIC group and 1.5 cm^2^ in the MFx group. For these sizes, MFx is still believed to be the most appropriate indications [5,6,7,14]. Volz et al. [27], in a prospective multicentre clinical trial, compared AMIC versus MFx on 47 patients. Similarly, they found a significant greater value of Cincinnati score and lower pain level in the AMIC cohort with an average size of the defects 3.6 cm^2^. However, differently to us, the MRI findings were significantly better in the AMIC group. They further compared glued versus sutured AMIC membrane—the glued membrane performed better than the sutured. Indeed, although suturing allows for greater membrane stability, it produces partial thickness lesions of the cartilage. These lesions may not heal, and may enlarge over the time [33,34]. Hunzinker et al. [35] demonstrated severe tissue impairment of the peri-suture area, which may lead to pain, reduced healing, and premature osteoarthritis [35]. It is unclear whether to use glue or no fixation. However, we believe that glue did not interfere negatively with cartilage regeneration and can be safely employed during AMIC. Similarly, Anders et al. [28] performed a comparative study between glued AMIC, sutured AMIC, and isolated MFx. The modified Cincinnati and ICRS scores were comparable between the three groups at two-year follow-up, as were the MRI findings. Regarding complications, while Anders et al. [28] reported no complications, Volz et al. [27] reported one total knee arthroplasty in the AMIC group and one revision in the MFx cohort. These results are in contrast to our findings, which reported significant lower rates of failure and revision surgery in the AMIC group. We hypothesise that the reason of this discrepancies arises from the longer follow-up of the present study (42.1 vs. 24 months). 

De Girolamo et al. [23] performed enhanced AMIC with bone marrow aspirate concentrate (BMAC) harvested form the ipsilateral iliac crest on 24 patients. They found comparability between AMIC and the enhanced technique at 9 years follow-up. However, given the faster recovery in the enhanced cohort, they recommended this procedure on patients who require faster return to sports [23]. In a similar setting involving nine patients, Enea et al. [36] found that BMAC-enhanced AMIC is safe and provides good clinical outcomes at 22 months follow-up. Additionally, they performed a second-look arthroscopy on five patients. This demonstrated a normal cartilage in one patient, nearly normal cartilage in three patients, and abnormal healing in one patient [36]. They further performed a biopsy in two patients, evidencing hyaline-like tissue, rich in proteoglycans and chondrocytes [36]. Given the simple and quick execution, enhanced AMIC may prove interesting for the management of chondral defects, and further investigations are required. 

Currently, there is a growing interest in new strategies for regeneration of chondral defects. Pipino et al. reported their preliminary results of MFx enhanced with the hydrogel polyglucosamine/glucosamine carbonate (PG/GC) in a clinical setting [37]. The Western Ontario and McMaster Universities Osteoarthritis Index (WOMAC) score of their patients improved of 88% at 6 months and 95% at 24 months [37]. Several other synthetic scaffolds have been proposed for MFx augmentation. Synthetic polymers, such as polyglycolic acid (PGA), polylactic acid (PLA), polylactic-co-glycolic acid (PLGA), and polyethylene glycol-terephthalate/polybutylene terephthalate (PEOT/PBT), have been investigated [38,39,40]. However, evidence is lacking and their use is still debated [41,42].

Patients presenting non-vital subchondral bone who received AMIC combined with cancellous bone grafting showed similar results compared to those who underwent the isolated chondral procedure. The cancellous bone used for the graft was harvested from the ipsilateral iliac crest. Given its osteogenic and neoangiogenic potential, iliac crest cancellous bone graft is rapidly incorporated into the host site [43,44]. However, the limited number of samples included for analysis may have influenced the results. Similar limitations can be inferred with regards to the comparison between the Chondro-Gide and Cartimaix membranes. Patients did not consent to be randomly allocated or blinded, representing an important limitation. The morphological quality assessment of regeneration was evaluated through the MOCART score, which evidenced no differences between the two techniques. Whether the morphological appearance at MRI is reliable in predicting clinical outcome after cartilage repair is questionable. The MRI classification demonstrates no correlation with clinical outcomes after cartilage repair surgery [45,46,47]. Older age is a negative prognostic factor for the success of chondral procedures [48,49,50]. The AMIC cohort was slightly younger than the MFx group. Although this difference was not statistically significant, we cannot estimate whether this may have affected our results. The limited number of the included patients jeopardises the capability to investigate uncommon complications. Longer follow-up is required to establish long-term complications and morphological changes in the quality of regenerated cartilage.

## 5. Conclusions

AMIC demonstrated superiority over MFx for focal unipolar chondral defects of the femorotibial joint of the knee. At approximately 40 months follow-up, IKDC, Lysholm, and VAS were greater in the AMIC group. Patients treated with AMIC also demonstrated a higher level of sporting activity, along with lower rates of failure and revision surgery.

## Figures and Tables

**Table 1:** Demographic data of the patients (n.s.: not significant).

Endpoint	AMIC (n = 52)	MFx (n = 31)	*p*
Follow-up (months)	43.7 ± 27.6	39.5 ± 19.1	n.s.
Age	29.5 ± 12.1	31.3 ± 9.9	n.s.
Gender (female)	34.6%	32.3%	n.s.
Side (*right leg*)	55.8%	54.8%	n.s.
Side (knee compartment)			
Lateral	35%	42%	n.s.
Medial	65%	58%	n.s.
Spongiosa transplantation	32.7%	-	
Membrane			
Cartimaix	23%		
Chondro-Gide	77%		
Symptom duration (months)	48.1 ± 80.7	35.3 ± 66.8	n.s.
Length of hospital stay (days)	4.5 ± 1.6	3.1 ± 0.9	0.03
Area of defect (cm^2^)	2.8 ± 2.5	2.6 ± 1.8	n.s.
BMI (kg/m^2^)	27.1 ± 4.4	26.5 ± 3.9	n.s.
Compartment			
Lateral compartment	34.6%	29.0%	n.s.
Medial compartment	65.4%	71.0%%	n.s.

**Table 2:** Results of scores.

Endpoint	AMIC (*n* = 52)	MFx (*n* = 31)	95% CI	MD	*p*
IKCD	75.9 ± 24.6	63.3 ± 6.3	3.619, 1.581	12.6	0.007
Lysholm	71.2 ± 24.3	59.9 ± 12.5	1.942, 20.658	11.3	0.02
MOCART	70.0 ± 19.4	68.4 ± 14.3	−6.384, 9.584	1.6	0.7
VAS (*0–10*)	2.5 ± 2.1	4.1 ± 3.3	0.422, 2.778	1.6	0.008
Tegner	4.8 ± 1.5	3.1 ± 0.9	1.108, 2.292	1.7	<0.0001

**Table 3:** Complications.

Endpoint	AMIC (*n* = 52)	MFx (*n* = 31)	95% CI	OR	*p*
Failure	3.8%	29.0%	0.0195, 0.4902	0.098	0.005
Knee arthroplasty	0	6.5%	0.0054, 2.5005	0.116	0.2
Revision surgery	1.9%	19.4%	0.0093, 0.7159	0.082	0.02

## Data Availability

Data is contained within the article or supplementary material.
